# Carbon Dots in Enantioselective Sensing

**DOI:** 10.3390/s24123945

**Published:** 2024-06-18

**Authors:** Martina Bortolami, Antonella Curulli, Paola Di Matteo, Rita Petrucci, Marta Feroci

**Affiliations:** 1 Department of Basic and Applied Sciences for Engineering, Sapienza University of Rome, 00161 Rome, Italy; martina.bortolami@uniroma1.it (M.B.); p.dimatteo@uniroma1.it (P.D.M.); rita.petrucci@uniroma1.it (R.P.); 2 Consiglio Nazionale delle Ricerche, Istituto per lo Studio dei Materiali Nanostrutturati, Unità Operativa di Supporto Sapienza, 00161 Rome, Italy; antonella.curulli@cnr.it

**Keywords:** chiral carbon dots, electrochemical sensors, fluorescence sensors, enantiorecognition

## Abstract

Chirality has a crucial effect on clinical, chemical and biological research since most bioactive compounds are chiral in the natural world. It is thus important to evaluate the enantiomeric ratio (or the enantiopurity) of the selected chiral analytes. To this purpose, fluorescence and electrochemical sensors, in which a chiral modifier is present, are reported in the literature. In this review, fluorescence and electrochemical sensors for enantiorecognition, in which chiral carbon dots (CDs) are used, are reported. Chiral CDs are a novel zero-dimensional carbon-based nanomaterial with a graphitic or amorphous carbon core and a chiral surface. They are nanoparticles with a high surface-to-volume ratio and good conductivity. Moreover, they have the advantages of good biocompatibility, multi-color emission, good conductivity and easy surface functionalization. Their exploitation in enantioselective sensing is the object of this review, in which several examples of fluorescent and electrochemical sensors, containing chiral CDs, are analyzed and discussed. A brief introduction to the most common synthetic procedures of chiral CDs is also reported, evidencing strengths and weaknesses. Finally, consideration concerning the potential challenges and future opportunities for the application of chiral CDs to the enantioselective sensing world are outlined.

## 1. Introduction

Chirality is an important property of many molecules used in all fields of chemistry. The definition for “Chirality” reported in the IUPAC Gold Book is “The geometric property of a rigid object (or spatial arrangement of points or atoms) of being non-superimposable on its mirror image” [1]. This property is particularly important in biologically active molecules, where the two enantiomers can have very different activities. The sadly well-known example of thalidomide [2] is only one of the many molecules whose enantiomers can have a positive and a negative effect on organisms. Among chiral biological molecules, amino acids are particularly important, being the constituents of proteins, enzymes, hormones, etc. Natural amino acids are generally L-enantiomers; nonetheless, recently, the presence of D-amino acids was put in evidence and they have been related to some disorders, such as kidney diseases [3] or neurodegeneration [4]. Their detection and quantitation are thus particularly important.

Nonetheless, sensors able to carry out the recognition of single enantiomers are not so common, apart from biosensors based on chiral biological molecules (enzymes, antigen–antibody, etc.) [5,6], whose most famous example is the amperometric glucose biosensor, based on glucose oxidase and catalase [7]. Moreover, the possibility of quantifying the enantiomeric excess (e. e.) when both enantiomers are present is of obvious interest and, to this aim, the use of chiral materials is necessary [8]. To this purpose, many different analytical techniques have been used, like high-performance liquid chromatography (HPLC) or gas chromatography (GC).

In order to enhance the sensing ability of various analytical techniques, many nanostructures have been considered, mainly due to their physico-chemical properties, among which the high surface-to-volume ratio is one of the most exploited [9,10].

Quantum dots are one-dimensional nanoparticles whose importance has been very recently underlined by the Nobel Prize in Chemistry 2023 to Moungi G. Bawendi, Louis E. Brus and Aleksey Yekimov for “the discovery and development of quantum dots. These tiny particles have unique properties and now spread their light from television screen and LED lamps. They catalyse chemical reactions and their clear light can illuminate tumor tissue for a surgeon” [11]. Carbon quantum dots (or simply carbon dots, CDs) are a sub-class of quantum dots, consisting of a carbon core (graphitic or amorphous) and various functional groups on the surface [12,13,14]. Their physico-chemical properties depend on the starting materials and on the synthetic methodology. In fact, it is possible to obtain CDs by both disrupting larger materials (top-down approach) and by polymerization/carbonization of small molecules (bottom-up approach). The synthesis and the study of the properties of CDs have been the object of many recent papers and we suggest referring to the literature for a deeper knowledge of such topics [15,16,17,18]. Besides the very high surface-to-volume ratio of CDs, photochemical and physico-chemical stability, biocompatibility and very often water solubility, their outstanding fluorescence ability [15,19,20] has recently made them nanomaterials of first choice for fluorescence sensors [12,21].

However, the chiral aspects of analytes are rarely taken into consideration and, very frequently, no mention of optical configuration is reported [22]. Enantiorecognition needs, of course, a chiral structure in the sensing device. The object of this review is the use, to this purpose, of chiral carbon dots or chiral composites containing CDs [23,24,25,26,27].

The aim of this paper is the review of the applications of chiral CDs to enantioselective sensing, enantiorecognition and enantiomeric excess evaluation, exploiting both the fluorescence properties of CDs and their ability to chirally modify electrodes with conductive nanoparticles. In the next paragraph, a brief overview of the synthesis of chiral carbon dots will be reported.

## 2. Synthesis of Chiral Carbon Dots

**Figure 1:**
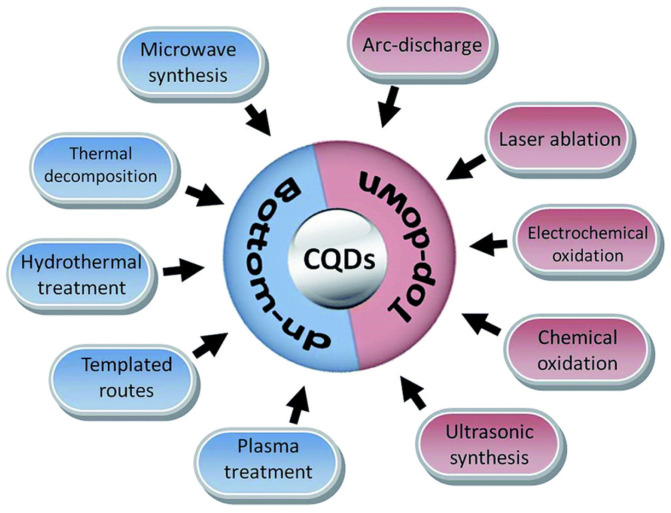
Schematic illustration of carbon quantum dots (CQDs) preparation via bottom-up and top-down approaches. Reproduced with permission from ref. [28]. Copyright 2017, Royal Society of Chemistry.

Chiral carbon dots can be obtained by both bottom-up and top-down synthesis. Moreover, to have surface chiral information, chiral starting material can be used (in the case of a bottom-up technique) or chiral post-functionalization of achiral CDs can be carried out (with both bottom-up and top-down techniques), whatever synthetic method is used (arch discharge, microwave synthesis, laser ablation, chemical or electrochemical oxidation, hydrothermal treatment, etc., [28], as shown in Figure 1).

### 2.1. Chiral Small Molecules as Starting Material (Bottom-Up Approach)

**Figure 2:**
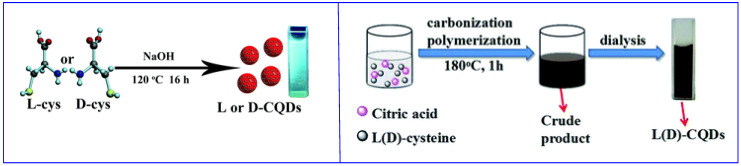
Schematic illustration of chiral carbon quantum dots (CQDs) preparation by hydrothermal method. (**Left**): using only one starting material. (**Right**): using two starting materials. Reproduced with permission from ref. [30]. Copyright 2016, Royal Society of Chemistry.

The bottom-up approach allows CDs synthesis by polymerization/carbonization of small molecules. The obtained CDs show on the surface the functional groups present on the starting materials. If the starting material is a chiral molecule, usually the chiral functionality is maintained on the surface of the nanoparticles. Often, hydrothermal synthesis is carried out, starting from one (Figure 2, left [29]) or two (Figure 2, right [30]) kinds of small molecules, at least one of which is chiral. It is also possible to obtain CDs by thermal carbonization (in the absence of solvents), microwave synthesis and electrochemical oxidation [28]. In all cases, most of the reagents are used to obtain the graphitic (or amorphous) carbon core.

The main drawback of this kind of synthesis is, thus, that a large amount of chiral starting material is used for the formation of the carbonized core of carbon dots (in which the chiral information is not necessary). This problem is overcome using chiral post-functionalization.

### 2.2. Large Carbon-Based Material Disgregation (Top-Down Approach)

The disaggregation of large carbon-based materials (graphite, polymers, etc.), by means of chemical or electrochemical oxidation, laser ablation, arc-discharge, etc. [12,28], usually leads to achiral carbon dots. This means that the chiral information is normally gained by surface chiral post-functionalization. The top-down approach is used less in CDs synthesis, possibly because of a less precise control in nanoparticle dimensions, which leads to less precise control of their physico-chemical properties.

### 2.3. Chiral Post-Functionalization

**Figure 3:**
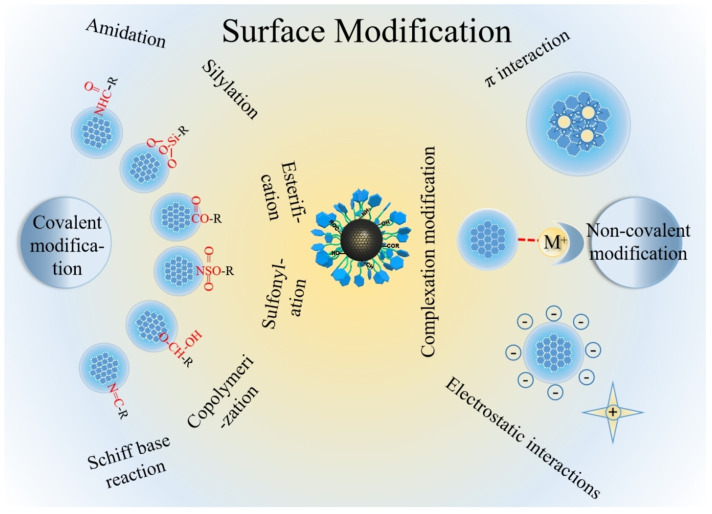
Schematic illustration of post-functionalization of carbon dots. Reproduced with permission from ref. [31]. Copyright 2023, Wiley-VCH GmbH.

In order to save on the amount of chiral reagent, the best way is to synthesize CDs using achiral molecules and then to exploit the surface functional groups to bind the chiral agent either by adsorption or by covalent bonds (Figure 3 [31]).

**Figure 4:**
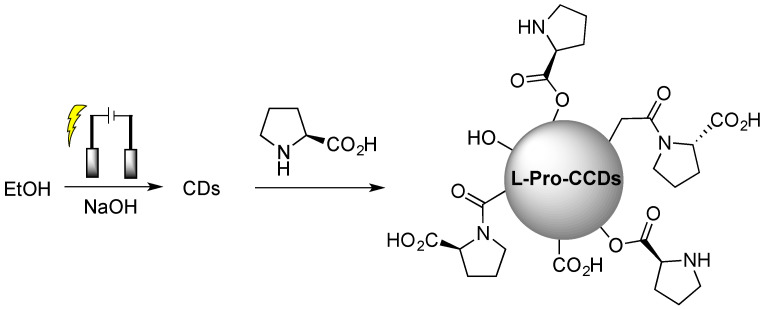
Schematic illustration of chiral carbon quantum dots (CQDs) preparation by chiral post-functionalization of electrochemically synthesized CDs from ethanol. Reproduced with permission from ref. [32]. Copyright 2022, MDPI.

Obviously, covalent functionalization guarantees higher stability of the chiral surface functional groups during utilization and, thus, the possibility to reuse such chiral CDs in subsequent runs, as reported in the case of electrochemically synthesized CDs from ethanol, subsequently covalently functionalized with L-proline (Figure 4 [32]), used for the enantioselective Aldol reaction.

## 3. Fluorescence and Colorimetric Chiral Sensors

As previously reported, one of the main characteristics of carbon dots is their fluorescence ability, which made them privileged nanoparticles in biomedicine and in fluorescence sensors. The fluorescence emission of CDs can be enhanced or quenched by the presence of the chiral analyte. In some cases, a change in color of the CDs solution is also observed, allowing for a dual-mode fluorescence/colorimetric sensor. The target molecules of such sensors are mainly amino acids but, in some cases, different chiral molecules (of biological importance) have been considered.

**Figure 5:**
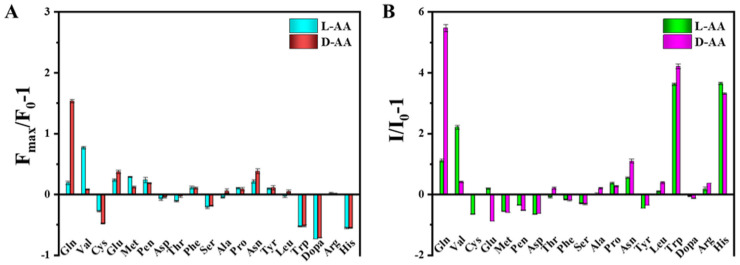
Fluorescent (**A**) and colorimetric (**B**) chiral recognition ability of TCDs. Reproduced with permission from ref. [33]. Copyright 2023, American Chemical Society.

Ren, Zhang and colleagues (Table 1, entry 1 [33]) reported a dual-mode, fluorescence/colorimetric sensor using carbon dots (TCDs) synthesized from *N*-methyl-1,2-benzene diamine dihydrochloride (OTD) and L-tryptophan (L-Trp) as a chiral agent by a hydrothermal method. The chirality of starting tryptophan was maintained in the CDs, as demonstrated by circular dichroism. Target analytes were amino acids and, in particular, L- and D-valine (L/D-Val) and L- and D-glutamine (L/D-Gln). The detection process was carried out in the presence of H_2_O_2_, which etched TCDs (their average size passed from 6.4 to 2.7 nm when treated with H_2_O_2_) and simultaneously reacted with the diamine, leading to a dimerization product. The effects of this process led to a preferential interaction with only one of the two enantiomers of amino acids. The interaction between the analyte and L-Trp on CDs surface is mainly due to hydrogen bonding. This selective interaction can lead to an increase in fluorescence emission (Figure 5A) and a change in solution color (Figure 5B). The best results were obtained with L/D-Gln and L/D-Val, whose enantiomers could be colorimetrically discriminated by the naked eye.

**Figure 6:**
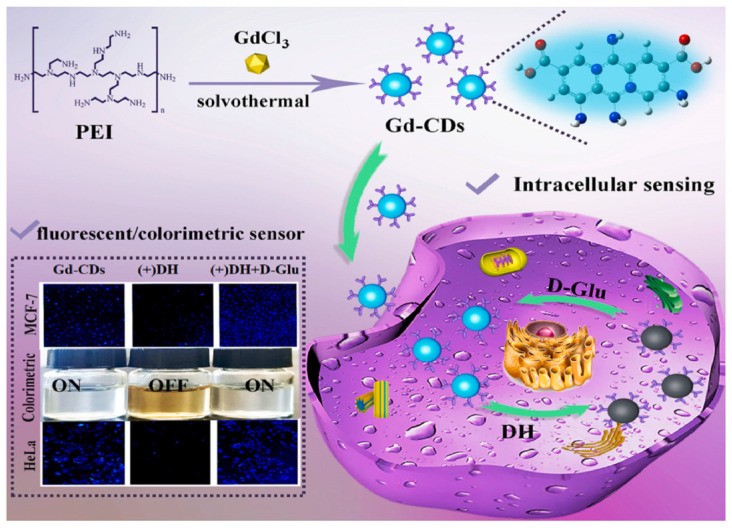
Gd-doped carbon dots as fluorescent/colorimetric dual-mode sensor for D-Glu recognition. Reproduced with permission from ref. [34]. Copyright 2023, Elsevier.

**Figure 7:**
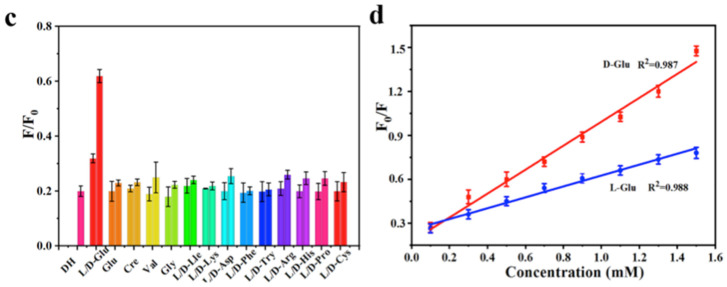
Fluorescence response of Gd-doped CDs to various amino acids (**c**) and different relationship concentration/fluorescence emission for D/L-Glu (**d**). Reproduced with permission from ref. [34]. Copyright 2023, Elsevier.

Sun, Jiang and colleagues (Table 1, entry 2 [34]) obtained a dual-mode fluorescence/colorimetric sensor with high sensitivity using gadolinium-(III)-doped CDs functionalized (non-covalently) with dopamine hydrochloride. In particular, Gd-(III)-doped CDs were synthesized by a top-down solvothermal method from a DMF solution of GdCl_3_ and polyetherimide (PEI) and post-functionalized with dopamine hydrochloride (DH). The presence of DH on the CDs surface quenched completely the fluorescence emission, while coloring the solution. Despite no chiral agent being used for this synthesis, the exposure of such Gd/DH carbon dots to the two enantiomers of glutamic acid (Glu) gave very different fluorescent and colorimetric effects (Figure 6). In particular, D-Glu was able to restore the fluorescence ability and color of CDs solution, while L-enantiomer was less effective. The authors hypothesized that DH interacted with the functional groups on the surface of CDs, with a quenching of fluorescence. The presence of D-Glu destroyed such complex structures, restoring the fluorescence ability (Figure 7, left). DFT calculations demonstrated that HOMO-LUMO interactions between DH and D-Glu were easier than with L-Glu. This is put in evidence by the very different slope of the calibration curves of the two enantiomers (Figure 7, right). Finally, the sensor was applied to real samples of human urine and mouse serum for determining L-Glu and the corresponding recoveries ranged from 93.70% to 122.00% in human urine and from 94.00% to 105.30% in mouse serum.

**Figure 8:**
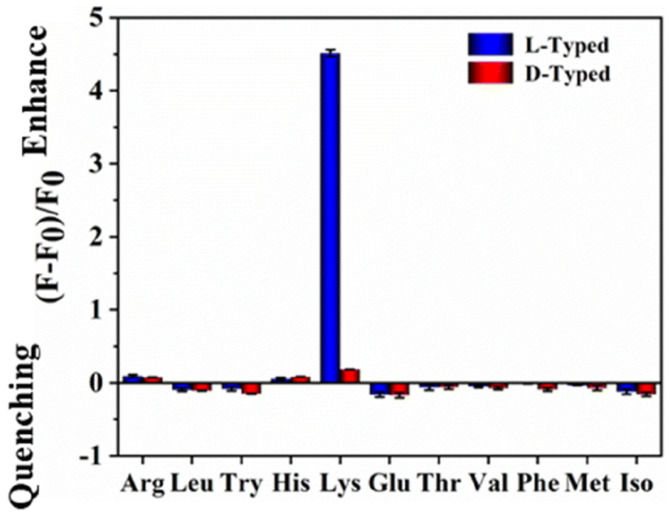
Fluorescence response of NADES-derived CDs to various amino acids. Reproduced with permission from ref. [35]. Copyright 2022, Royal Society of Chemistry.

Chiral natural deep eutectic solvents (NADES) were used as starting material, along with vine tea, for the hydrothermal synthesis of CDs by Xia, Huang and colleagues (Table 1, entry 3 [35]). NADES was obtained using choline chloride and D-(-)-fructose as the chiral agent. The obtained CDs showed a high quantum yield and only L-Lysine (L-Lys), among amino acids, had a remarkable enhancement on the fluorescence of CDs (Figure 8) also in the presence of other amino acids. Moreover, this sensor is sensitive and it has a remarkable limit of detection of 10 nM for L-Lys. The authors report that only L-Lys is able to accept the excited electrons of the carbon dots, thus avoiding the recombination between electrons and holes, yielding the high fluorescence signal (Figure 8). Real samples of urine, human serum and energy drinks were analyzed for quantifying L-Lys and the corresponding recoveries, ranging from 86.19% to 109.65%, were considered satisfactory. 

**Figure 9:**
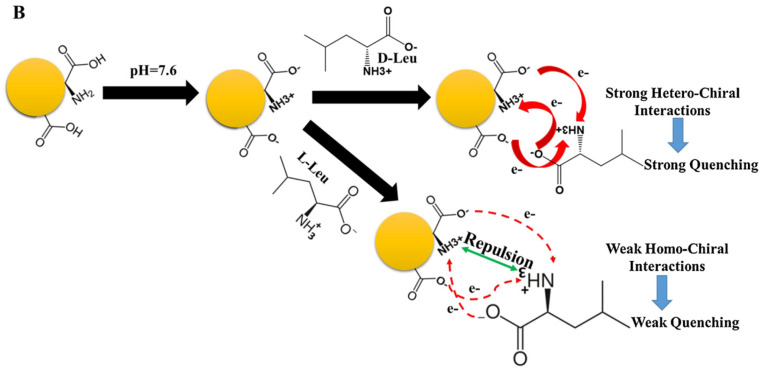
Proposed mechanism for the enantioselective determination of D/L-Leu. Reproduced with permission from ref. [36]. Copyright 2021, Elsevier.

Akhond and colleagues (Table 1, entry 4 [36]) reported an ultrasensitive fluorescence sensor for the detection of both enantiomers of leucine (L/D-Leu). In this case, CDs were obtained by simple thermal treatment of L-Glu in the absence of any solvent. Such CDs have good fluorescence ability, which is selectively quenched by leucine (more efficiently by D-Leu) (Figure 9). Such a method is efficient also in the presence of other amino acids as interferents. The limit of detection was 1.7 nM for D-Leu and 20.0 nM for L-Leu. Strong hetero-chiral interactions between D-Leu and the CDs surface functional groups due to L-Glu accounted for such selectivity, as reported in Figure 9. D-Leu was then determined in real samples of body-building supplements and human serum, obtaining good results in terms of recoveries. In fact, for the body-building supplements, they ranged from 96.10 to 102.40% and, for human serum, from 95.00% to 103.3%.

The use of enzymes as a highly specific chiral detector is well known and widely exploited (e.g., in the amperometric glucose biosensors). The specificity of the enzyme for its substrate usually allows for highly sensitive sensors, with negligible interference by other similar molecules. Wang, Men and colleagues (Table 1, entry 5 [37]) reported a fluorescence-colorimetric biosensor for D-glucose, in which the recognition element is the enzyme D-glucose oxidase (GOX), using CDs as fluorophores. CDs were obtained by hydrothermal synthesis from citric acid and cysteine. The very high selectivity of the enzyme for D-glucose allowed for the production of H_2_O_2_ when in the presence of D-glucose. Hydrogen peroxide mediated the in situ formation of Au nanoparticles (from Au seeds present in the solution). These Au nanoparticles quenched the CDs fluorescence. The presence, in solution, of the L-enantiomer did not affect the measure. It is thus clear that such a biosensor is not able to quantify an enantiomeric excess, but it can be used to determine D-glucose in real blood samples; the obtained results were comparable with those coming from a commercial glucometer.

A very similar approach was described by Ji, Li and colleagues (Table 1, entry 6 [38]). In fact, they reported a fluorescence biosensor for D-alanine (D-Ala), in which the recognition element is another enzyme, i.e., D-α-amino acid oxidase (DAAO), using as a fluorophore CDs grafted on cellulose paper. Achiral CDs were obtained by microwave synthesis from citric acid and urea. The very high selectivity of the enzyme for the target amino acid allowed for the production of H_2_O_2_ when in the presence of D-Ala. As in the previous example, H_2_O_2_ mediated the formation of AuNPs quenching the fluorescence of CDs and the presence of L-Ala in solution did not affect the measure. Consequently, such a biosensor is not able to quantify the enantiomeric excess. This biosensor was used for determining D-Ala in human serum, in artificial gastric fluid and in cells of gastric cancer (BGC-823) and the corresponding recoveries were in the range from 94.20% to 106.60% in human serum, from 97.40% to 98.20% in artificial gastric fluid and from 94.40% to 106.80% in BGC-823 cells.

**Figure 10:**
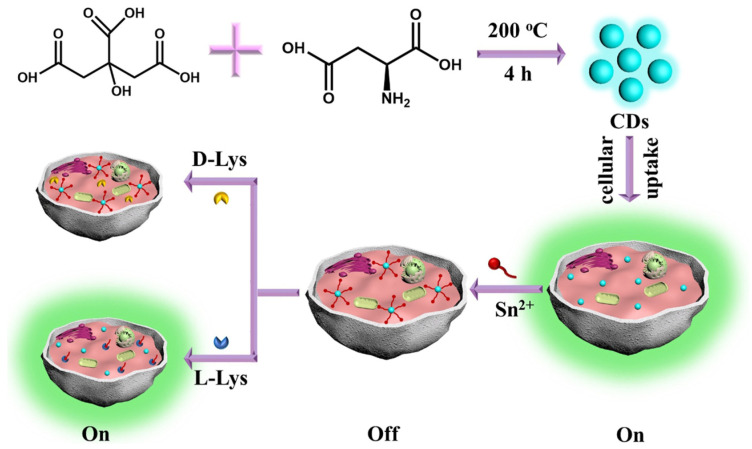
Schematic illustration of chiral CDs fabrication used for L-Lys assay in the presence of Sn^2+^. Reproduced with permission from ref. [39]. Copyright 2020, Elsevier.

Xie, Zheng and colleagues (Table 1, entry 7 [39]) reported the bottom-up synthesis of chiral CDs by a hydrothermal method starting from citric acid and L-aspartic acid (L-Asp) as the chiral agent. These CDs were highly fluorescent and the co-ordination of Sn^2+^ quenched their fluorescence. Such a fluorescence on–off effect was reversed (with high selectivity and excellent sensitivity) by the presence of L-Lys as a competitive substrate in binding CDs (Figure 10). The authors applied such a sensor to the intracellular detection of L-Lys.

**Figure 11:**
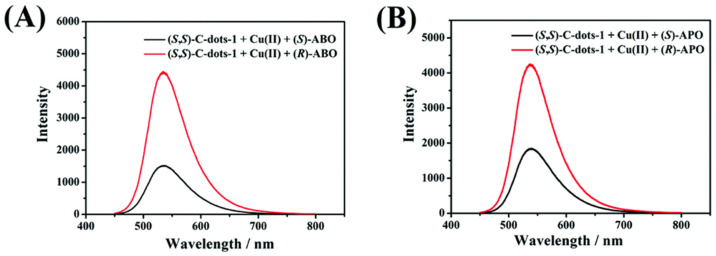
Different fluorescence response of chiral CDs to amino alcohols enantiomers. Reproduced with permission from ref. [40]. Copyright 2020, Royal Society of Chemistry.

**Figure 12:**
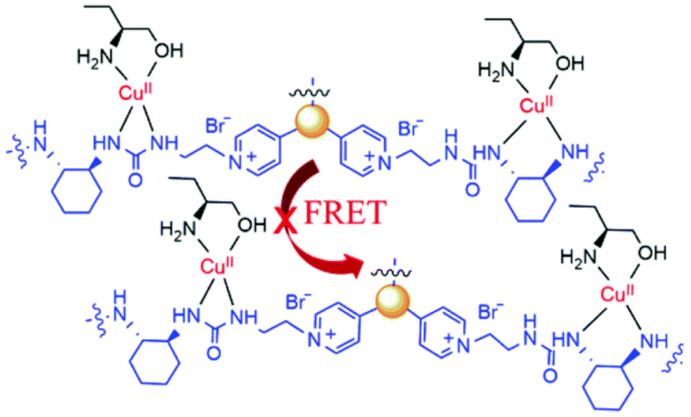
Schematic illustration of enantioselective quenching due to S-ABO. Reproduced with permission from ref. [40]. Copyright 2020, Royal Society of Chemistry.

Chiral carbon dots able to enantioselectively discriminate nonaromatic amino alcohols were reported by Kong and colleagues (Table 1, entry 8 [40]). In particular, post-functionalization of achiral fluorescent CDs with (1*S*,2*S*)-1,2-diaminocyclohexane allowed a selective interaction with 2-aminobutan-1-ol (ABO) and 2-aminopropan-1-ol (APO) in the presence of Cu^2+^ (Figure 11), leading to fluorescence quenching as the metal cation permits an increase in the distance between two carbon dots, thus rendering a more difficult transfer of the fluorescence resonance energy (FRET). In particular, the stronger interaction with *S*-isomers (Figure 12) allowed a good enantiorecognition, although in a small concentration range.

**Figure 13:**
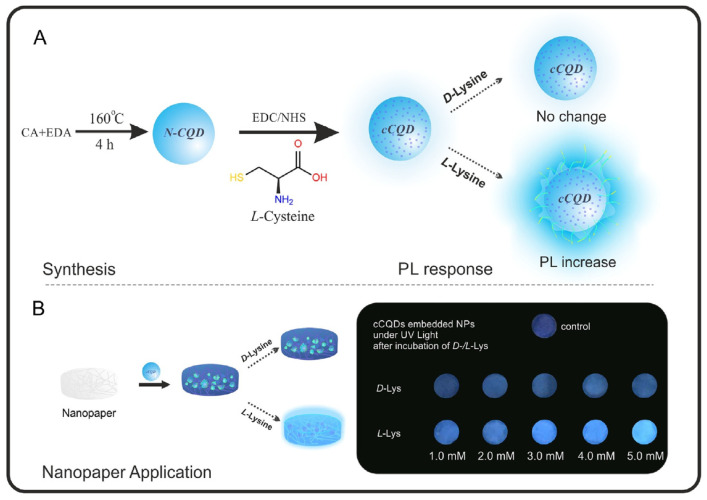
Schematic illustration of chiral CDs synthesis and enantioselective fluorescent emission enhancement in the presence of L-Lys. Reproduced with permission from ref. [41]. Copyright 2019, Elsevier.

Bingol and colleagues (Table 1, entry 9 [41]) were able to obtain a selective chiral fluorescence sensor for L-Lys, embedding chiral CDs onto a paper sheet. Chiral CDs were obtained by covalent post-functionalization with L-cysteine (L-Cys) of achiral CDs. The ability of L-Lys to enhance the fluorescent emission of such a system (Figure 13) allowed for the quantification of the enantiomeric excess in the range of 10–100% of L-Lys. The enhancement in fluorescence emission is probably due to an interaction between the analyte and the carbon dot surface groups (due to L-Cys), which hinder the relaxation of the excited states.

The possibility of creating a tridimensional chiral porous organic cage by reaction of 1,3,5-triformylbenzene and (1*R*,2*R*)-1,2-diaminocyclohexane (imine bonds) and decorating it with CDs was exploited by Li, Zhang and colleagues (Table 1, entry 10 [42]). The presence of carbon dots gave a good fluorescence ability to such a nanocomposite, while the starting amine rendered the pores of the organic cage chiral. Such a system was able to carry out the enantiorecognition of chiral alcohols. In particular, D-/L-phenylalaninol (PA) and R-/S-phenylethanol (PE) were considered. The two enantiomers of each chiral alcohol, binding the CDs surface functional groups, quenched the native fluorescence to a different extent, rendering possible enantiorecognition.

**Figure 14:**
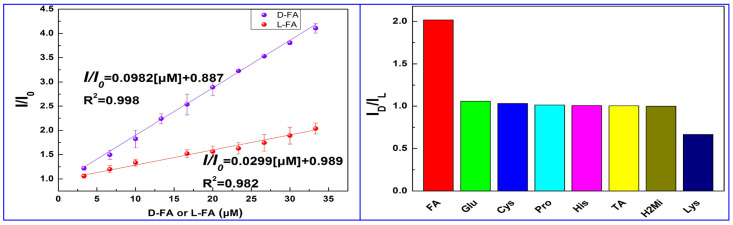
(**Left**): linear relationship of CDs fluorescent emission with different concentrations of D- and L-folic acid. (**Right**): CDs fluorescence intensity ratio I_D_/I_L_ with the addition of various chiral substrates. Reproduced with permission from ref. [43]. Copyright 2023, American Chemical Society.

Ding and colleagues (Table 1, entry 11 [43]) encapsulated CDs into metal-organic frameworks (MOFs) in order to have enantiorecognition of D- and L-folic acid (FA) with a fluorescence sensor. CDs were obtained by a hydrothermal bottom-up method from citric acid and L-Cys as the chiral agent and were encapsulated into a zeolitic imidazolate framework, forming a chiral composite used as a selective and sensitive “turn-on” fluorescent sensor, with a low detection limit (0.31 μM) for L-FA. The highly different fluorescence behavior of this system in the presence of the two enantiomers of folic acid is evident in Figure 14 (left), having a very different slope in the calibration curves. The high sensitivity towards FA is shown in comparison with the behavior of other analytes (Figure 14, right), mainly amino acids. The role of CDs was due to their fluorescence ability and selective binding of the analyte by means of surface functional groups. Next, the sensor was applied to real samples of folic acid supplements for determining L-FA, obtaining satisfactory recoveries in the range from 91.20 to 99.38%.

A multifunctional fluorescent sensor array for L/D-Cys enantiorecognition was reported by Zhang and colleagues (Table 1, entry 12 [44]). Such a sensor is constituted by supraparticles (SPs), obtained by the aggregation (via π–π stacking interactions) of 5,10,15,20-tetra(4-carboxyphenyl)porphyrin (TCPP), CDs (by hydrothermal synthesis from folic acid and polyetherimide) and Cu^2+^. The presence of two distinct fluorescence emission peaks (at 470 and 668 nm) for such SPs allowed study of the quenching ability of analytes at these two wavelengths. The metal cation forms a nonfluorescent complex with TCPP/CDs (static quenching effect), which can be destroyed (restoring fluorescence) by addition of chiral thiols. The combination of the results permitted the enantiorecognition of L- and D-Cys.

**Figure 15:**
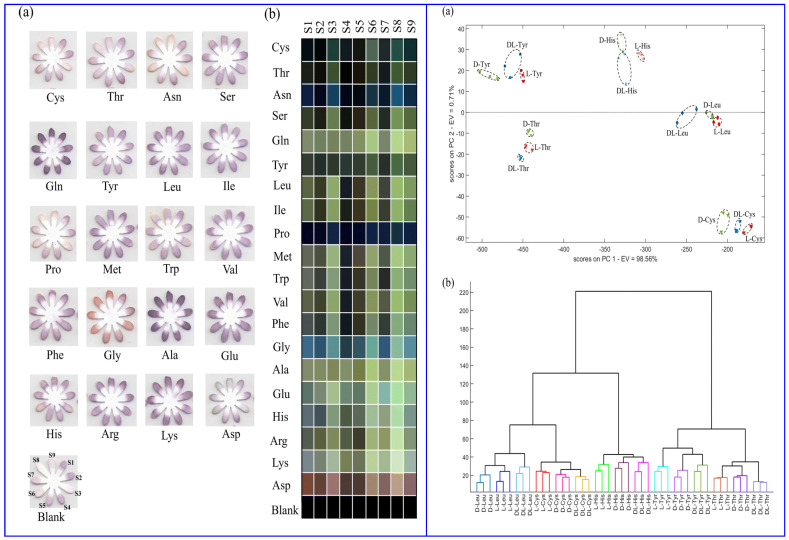
(**Left**): images of optical tongue before and after exposure to amino acids (**a**) and corresponding colorimetric difference maps (**b**). (**Right**): 2D principal component analysis (PCA), linear discriminant analysis (LDA) (**a**) and hierarchical cluster analysis (HCA) plot (**b**) for L-, D-, racemic amino acids. Reproduced with permission from ref. [45]. Copyright 2023, Royal Society of Chemistry.

Hemmateenejad and colleagues (Table 1, entry 13 [45]) reported an interesting colorimetric device (optical tongue) formed by an array of microfluidic sensors embedding metal-doped CDs. Such CDs were obtained by hydrothermal treatment of bovine serum albumin (BSA) and various transition metal salts. The different colorimetric response of the various sensors of the array at the exposure of different amino acids was analyzed by statistical and chemometric methods and allowed not only to discriminate between the 20 natural amino acids but also between enantiomers of the same amino acid (Figure 15). 

Cao and colleagues [46] reported a fluorescence enantiomeric recognition using CDs obtained by a hydrothermal method from 2,2-di(prop-2-yn-1-yl)malonic acid and cyclohexane-1,2-diamine in the presence of Hg^2+^. No chiral information about the starting diamine and no demonstration of chirality of the obtained CDs are reported. Moreover, the authors claimed an enantiomeric recognition of L/D-Trp and L/D-tartaric acid but the increase in fluorescence emission of CDs due to the presence of such analytes is really small and, moreover, the behavior of the two enantiomers is very similar. In fact, no plot of fluorescence emission vs. concentration in order to check the linearity of the response is reported, rendering this method not suitable for enantiomeric recognition.

As regards florescence/colorimetric sensors, chiral CDs have usually a dual role: to impart fluorescence ability to the solution and to selectively bind (mainly by hydrogen bonding) the analyte. In most cases, the fluorescence is quenched by analyte–CDs interaction but, in some examples, it is enhanced due to a hindrance in the relaxation step. In all cases, the chirality of CDs surface is crucial for the enantiorecognition.

As final considerations, LOD values achieved by the fluorescence/colorimetric sensors were described, μM to mM, and the corresponding linearity ranges seem to be wide, considering the application field.

Few sensors have been applied to real samples and a comparison with standard methods is generally missing.

Analytical performances of the reported fluorescent/colorimetric sensors based on CDs for enantiorecognition are reported in Table 1.

## 4. Electrochemical Chiral Sensors

When considering electrochemical sensors for enantiodiscrimination, CDs can be used as chiral agents and/or to enhance the electrode surface (due to their very high specific surface area) and conductivity (for their excellent electron transport capacity). In this case, CDs dimensions are usually less important, being responsible for the nanoparticles’ energy levels and, thus, for their fluorescence ability, while the composition of the CDs core (graphitic or amorphous carbon) is essential in electrochemical applications and responsible for their electrical conductivity. Preferential chiral analytes were tryptophan and tyrosine enantiomers, which are among the very few electroactive amino acids. The functionalization of the electrode surface with a chiral selector allows for a different electrochemical response (peak voltage and current) of the two enantiomers, depending on the different binding mode or inclusion mode in the case of inclusion complexes as in the presence of cyclodextrins. Usually, by reporting a calibration curve of peak current vs. enantiomeric excess (at a fixed concentration of the calibration curve), the unknown enantiomeric excess of a mixture of enantiomers can be determined.

**Figure 16:**
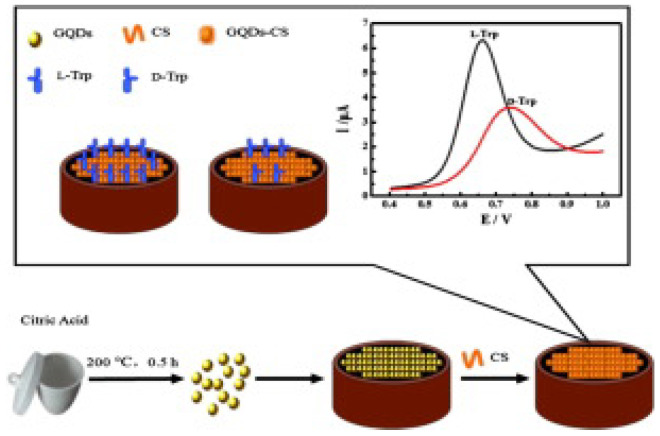
Schematic illustration for fabrication of a chiral sensor for tryptophan. Reproduced with permission from ref. [47]. Copyright 2015, Elsevier.

Among different chiral selectors, chitosan, a biocompatible polysaccharide, was chosen in two different papers for the detection of tryptophan. Kong and colleagues (Table 2, entry 1 [47]) described the use of a CDs–chitosan composite for the electrodeposition of a film on a glassy carbon electrode (GCE). Achiral carbon dots were obtained by a bottom-up approach starting from citric acid. Such a modified electrode was used for the enantiorecognition of Trp enantiomers, as demonstrated in the voltametric analysis reported in Figure 16. In fact, the different binding modes of the two enantiomers with the chiral agent (chitosan) produced a different electrochemical response in terms of peak potential and peak current. Nonetheless, no calibration curve for the determination of the enantiomeric ratio was reported, indicating only the potentiality (and not the practical application) of this electrochemical sensor.

**Figure 17:**
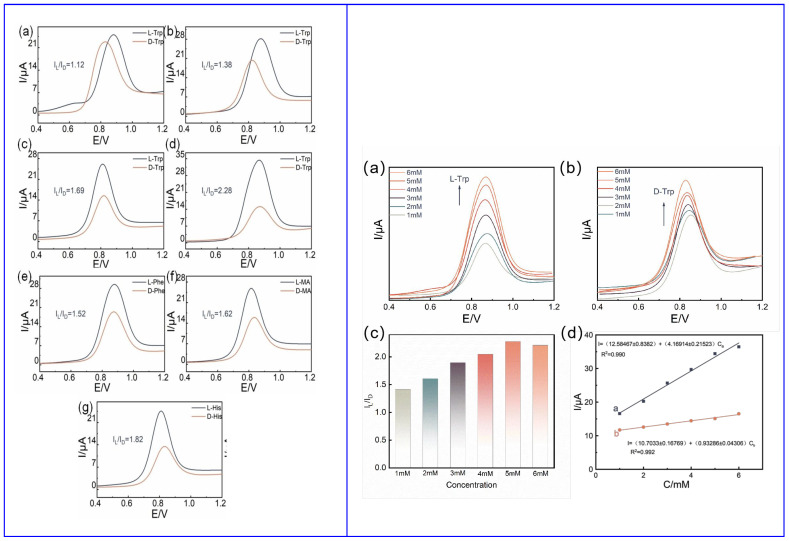
(**Left**): (**a**) CDs on GCE, (**b**) CS on GCE, (**c**) noncovalent CDs and CS on GCE, (**d**) covalent CDs–CS on GCE results for Trp enantiorecognition; (**e**) covalent CDs–CS on GCE results for Phe enantiorecognition; (**f**) covalent CDs–CS on GCE results for mandelic acid enantiorecognition; (**g**) covalent CDs–CS on GCE results for His enantiorecognition. (**Right**): CDs–CS on GCE performance on Trp enantiorecognition: (**a**) peak current vs. L-Trp concentration, (**b**) peak current vs. D-Trp concentration, (**c**) I_L_/I_D_ current ratio at different concentrations, (**d**) linearity range. Reproduced with permission from ref. [48]. Copyright 2023, Wiley.

Also, Wang and colleagues (Table 2, entry 2 [48]) used achiral carbon dots to enhance the electron transport ability of the system, based on differential pulse voltammetry (DPV). Again, the chiral selector was chitosan (CS). The authors explored two different modes of bonding between CS and CDs, covalent and noncovalent. Achiral CDs were, obviously, not able to discriminate between the two enantiomers of tryptophan but were able to enhance the current response in comparison to the bare electrode (GCE), due to the enhancement of its surface area. The higher stability of covalently bonded CDs–CS allowed them to be used for the enantiorecognition of tryptophan, histidine, phenylalanine and mandelic acid, through selective intermolecular hydrogen bonding. The different DPV response of enantiomers of the analytes, demonstrated in Figure 17 (left), allowed for calibration curves with very different slopes (Figure 17, right) and, thus, potentially, for enantiorecognition. Nonetheless, no quantitation measurements were reported.

D and L enantiomers of tartaric acid (Tart) gave different electrochemical responses in both linear sweep voltammetry (LSV) and electrochemical impedance spectroscopy (EIS) when using cysteine-derived carbon dots to modify a carbon paste electrode (Table 2, entry 3 [29]). Huang, Liu, Kang and colleagues reported a stronger interaction between the chiral selector and the analyte with the same configuration. Unfortunately, no calibration curve nor experiments for enantioselection were reported, so not verifying the potentiality of such a device.

**Figure 18:**
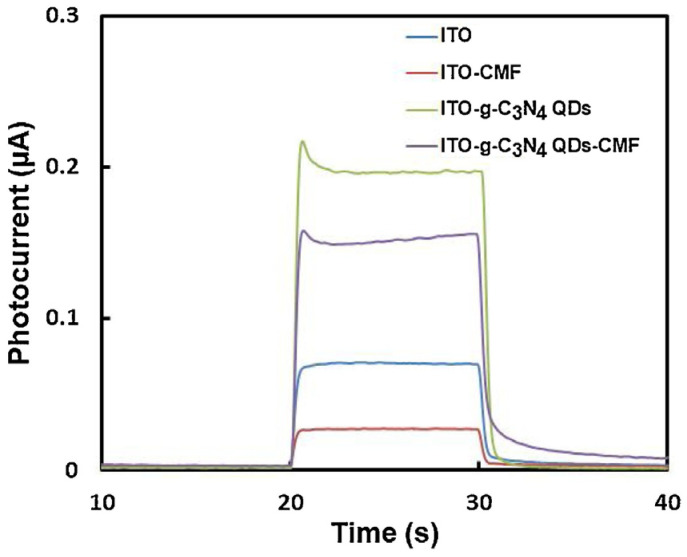
Photoelectrochemical responses for solutions of thyroxine. Reproduced with permission from ref. [49]. Copyright 2015, Elsevier.

Cong and colleagues (Table 2, entry 4 [49]) reported an efficient photo-electrochemical sensor based on the supramolecular interaction between a macrocyclic compound (chiral multifarene, CMF) and the hormone drug thyroxine (L-T_4_). In this case, graphitic carbon nitride CDs (achiral, obtained by thermal polymerization of melamine) were used to obtain a good photocurrent upon irradiation and thus improve the performance of the photo-electrochemical sensor. The presence of the chiral selector allowed discrimination between the two enantiomers of thyroxine, while the presence of CDs enhanced the photo-electrochemical response on ITO transparent electrode (Figure 18), due to the peculiar molecular orbital energies of these nanoparticles. The high specificity of the interaction between the chiral selector and analyte allowed precise measurements also in the presence of various interferents, such as different amino acids. As for the majority of biosensors, the limit of detection is very low, in the order of pM. L-T_4_ was determined in human serum samples (recoveries from 98.60% to 104.00%) and in L-T_4_ commercial tablets (recoveries from 94.96% to 97.65%).

**Figure 19:**
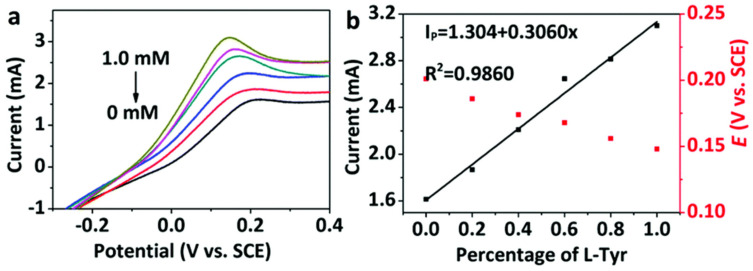
(**a**) CVs of Tyr enantiomers at different enantiomeric excesses using CDs on MOF and (**b**) linear relationship between current and L-Tyr percentage. Reproduced with permission from ref. [50]. Copyright 2020, Royal Society of Chemistry.

Kuang and colleagues (Table 2, entry 5 [50]) prepared a modified electrode by electrodeposition of chiral CDs on a metal-organic framework (MOF) on nickel foil. Carbon dots were obtained by bottom-up microwave pyrolysis of sorbitol, which acted as a chiral selector. The cyclic voltametric technique allowed registering different electrochemical responses (peak current and potential) for the two Tyr enantiomers and a linear relationship was obtained between the current and the percentage of L-isomer in the mixture (Figure 19, CVs and plot of the peak currents of the two enantiomers). Moreover, such a modified electrode resulted as stable for two weeks, which allows (potentially) for fabrication and later use.

The chiral agent in electrochemical sensors can be a chiral cavity, selectively forming an inclusion complex with the analyte. This is the case when β-cyclodextrins are used, as in the following examples.

**Figure 20:**
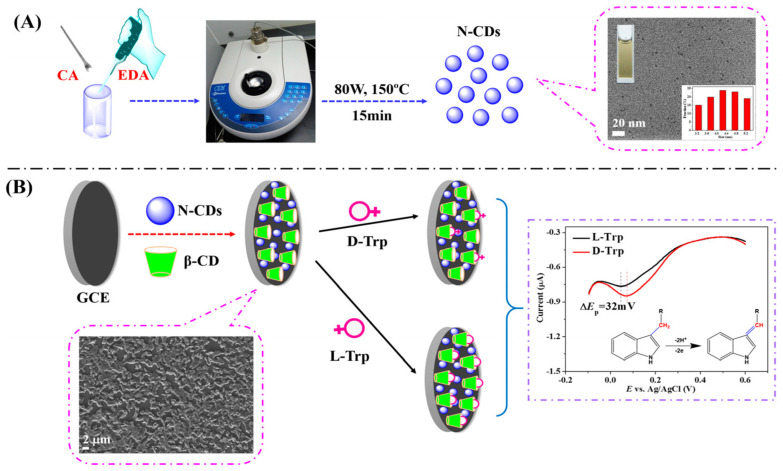
Schematic illustration of (**A**) chiral carbon quantum dots (CDs) preparation and (**B**) electrode assembly for Trp enantiorecognition. Reproduced with permission from ref. [51]. Copyright 2016, MDPI.

Huang and colleagues (Table 2, entry 6 [51]) described an efficient electrochemical sensor for the enantiorecognition of L/D-Trp based on a composite including CDs and β-cyclodextrin (no covalent bond). Such composite was electrodeposited on a GCE and used for the determination of Trp enantiomers by means of DPV. The potential peak difference was 32 mV and the enantioselectivity coefficient (I_L_/I_D_) was 0.90, due to the different binding interaction in the formation of the inclusion complexes. A linear correlation between the peak current and the percentage of L-Trp in a mixture of both enantiomers allowed an efficient determination of the enantiomeric excess at the total concentration of 5.0 mM. Figure 20, in the upper part (A), schematically describes the CDs bottom-up synthesis and size distribution, while the electrode fabrication and the voltametric response of the two Trp enantiomers are reported in the lower part (B) of the figure.

**Figure 21:**
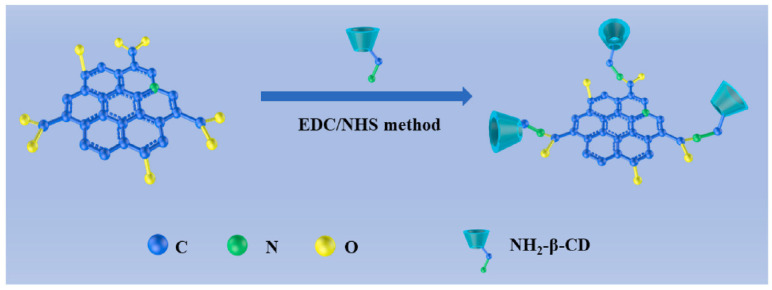
Synthesis of β-cyclodextrin functionalized CDs. Reproduced with permission from ref. [52]. Copyright 2021, Elsevier.

**Figure 22:**
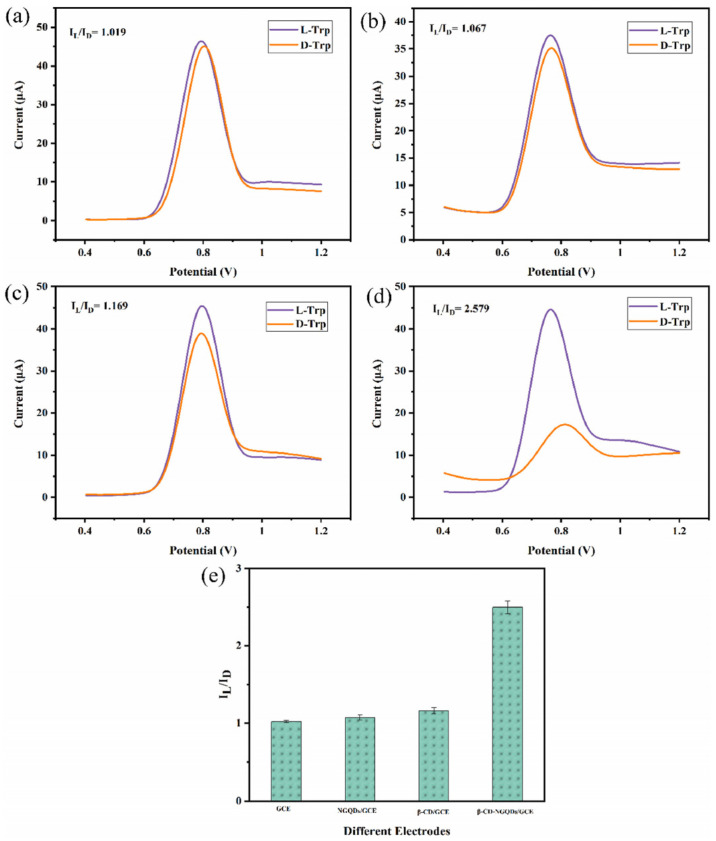
DPV of Trp enantiomers solutions on (**a**) GCE, (**b**) GCE functionalized with CDs, (**c**) GCE functionalized with β-cyclodextrin, (**d**) GCE functionalized with CDs covalently bonded to β-cyclodextrin and (**e**) histogram of the results. Reproduced with permission from ref. [52]. Copyright 2021, Elsevier.

An electrochemical sensor for tryptophan enantiomers was also reported by Mo and colleagues (Table 2, entry 7 [52]), functionalizing a GCE, by drop casting, with nitrogen-doped graphene quantum dots (β-CD-NGQDs). In this case, CDs were obtained by a top-down methodology starting from reduced graphene oxide (RGO), oxidized by nitric acid and covalently functionalized with β-cyclodextrin (Figure 21), which acted by chiral selector. The DPV analysis of L or D-Trp evidenced a very different behavior, mainly as regards the peak current intensity (see Figure 22 for a comparison between L and D-Trp DPV behavior on bare GCE (a), CDs functionalized GCE (b), β-cyclodextrin functionalized GCE (c), and β-cyclodextrin-CDs functionalized GCE (d)). An I_L_/I_D_ ratio of 2.57 was obtained. Unfortunately, no information about linearity range or limit of detection was reported. A theoretical study on the selective binding mode between the chiral cavity of cyclodextrin and the L-enantiomer was also reported.

Also, Huang and colleagues (Table 2, entry 8 [53]) used β-cyclodextrins covalently bonded to graphene-derived CDs for functionalizing a GCE by electrodeposition. Despite the higher selectivity of this chiral selector for D-Trp (with respect to L-Trp), the detection of the two enantiomers was reported only in solutions containing only one isomer at a concentration in the order of μM.

**Figure 23:**
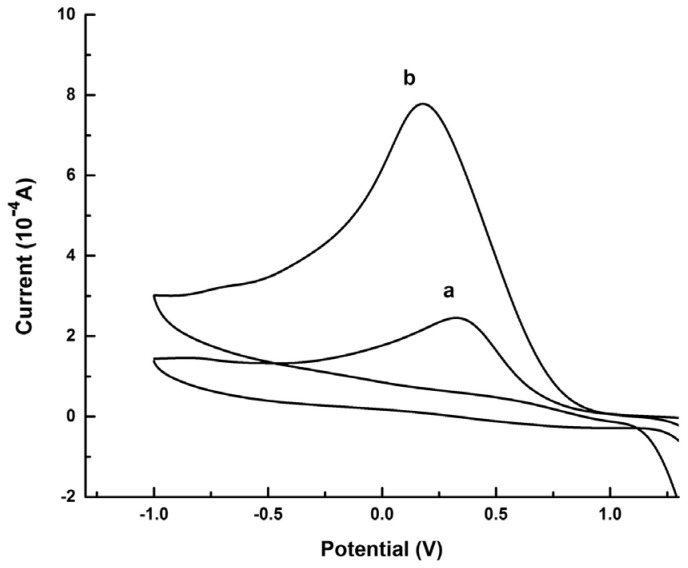
CVs of Tyr enantiomers on a GCE functionalized with β-cyclodextrin covalently bonded to graphene-derived CDs. (a): D-Tyr, (b): L-Tyr. Reproduced with permission from ref. [54]. Copyright 2017, Elsevier.

**Figure 24:**
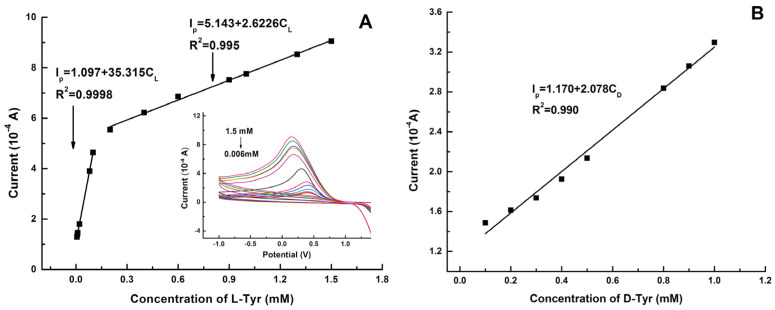
Linear diagram of the concentration of L-Tyr (**A**) and D-Tyr (**B**) in solution and the oxidation peak current of CV of a GCE functionalized with β-cyclodextrin covalently bonded to graphene-derived CDs. Reproduced with permission from ref. [54]. Copyright 2017, Elsevier.

Graphene quantum dots and β-cyclodextrins composites were used by Lu, Zhao and colleagues for the electrochemical determination of tyrosine (Tyr) enantiomers (Table 2, entry 9 [54]). CDs were obtained by acid oxidation of graphene and mixed with β-cyclodextrins. The resulting noncovalent mixture was then used for the electrodeposition on a GCE. Such a chiral modified electrode was applied for efficient enantiorecognition of L- and D-Tyr. In fact, the CV response was very different for D- and L-Tyr (Figure 23): L-Tyr yielded a higher peak current, due to the preferential interaction with the chiral selector. Two different linearity ranges were determined for L-Tyr (Figure 24A), allowing a very low detection limit (6.07 nM). Moreover, a linear correlation between the enantiomeric excess and the peak current was obtained at the total concentration of 1 mM (Figure 24B). To investigate the feasibility of this Tyr sensor’s applications to real samples, several serum samples of healthy people and depressed patients were analyzed. In fact, depression seems to be correlated to a deficiency of L-Tyr. The normal serum level of Tyr was 33–83 μM [54]. The serum Tyr concentrations of healthy people (41.75–95.67 μM) and of depression patients (32.45–64.55 μM) are in the normal range, but it is to be underlined that the concentration range of depression patients was lower than in healthy people, which was consistent with the hypothesis [54] that lower L-Tyr levels in humans can be correlated with the depression.

As previously stated, the main uses of CDs in this kind of electrochemical sensor are related to the good electrical conductivity of these nanoparticles and to their high surface-to-volume ratio, which allow for a more intense response. Moreover, their chiral surface permits a selective binding with one enantiomer of the analyte, allowing for a different device response and for the enantiorecognition. As a final comment, often in the described studies, the analytical performances of the sensors seem not to be analyzed with sufficient accuracy. In fact, LOD values and the corresponding linearity ranges are not reported in many examples and the application of the sensors to real samples has not been addressed generally.

The analytical parameters of the reported electrochemical sensors based on CDs for enantiorecognition are reported in Table 1.

## 5. Conclusions and Perspectives

In this section, some considerations and comments regarding the role of CDs and the different types of sensors are summarized. Finally, critical issues, challenges and future perspectives on enantioselective sensing are introduced.

The most used CDs synthetic approaches were hydrothermal and thermal, both being bottom-up methods, probably because they are well-established synthetic procedures. In a few cases, a top-down approach was included, in particular considering the examples involving β-cyclodextrin as a chiral agent. The bottom-up approach is the preferred one, most probably as it ensures higher control in particle dimension distribution and, consequently, physico-chemical properties [55]. In particular, both the CDs inner core structure and surface functional groups contribute to the performance of such particles. In fact, the chemical structure (and the dimensions) influences the molecular orbital energy levels of carbon dots, thus defining both the fluorescence and the electrochemical behavior (conductivity) [56].

How is chirality introduced in CDs and used for enantioselective sensing? Starting from a CDs bottom-up synthesis, the chirality is introduced preferentially involving polymerization or carbonization of small chiral molecules. The CDs surface was functionalized with chiral moieties but it is to be evidenced that a large amount of chiral material was lost during the synthesis procedure. It would be thus preferable to use chiral post-functionalization, but, among the reported studies, only three examples involved this approach [34,40,41]. Moreover, chiral post-functionalization allows for a larger choice in the chiral agent (not limited to cheap and abundant natural products), thus widening the number and nature of analyte targets of the sensing process.

Different chiral agents are employed, such as amino acids, organic acids, enzymes such GOX, sugars such as sorbitol, alcohols, amines, and natural polymers such as chitosan and β-cyclodextrin, among others, so versatile electrochemical and fluorescent chiral sensors are assembled. It is clear that the research strategies for designing novel chiral selectors with relatively higher enantioselectivity and reusability together with studies on chiral recognition mechanisms still need to be implemented.

Due to the high fluorescence ability of carbon dots, fluorescence sensors are often based on fluorescence quenching by an interaction between the analyte and CDs, thus allowing identification and quantitation of the selected enantiomer by an enantioselective quenching. 

As regards the electrochemical sensors, usually the peak potential difference for the two enantiomers is not large enough to allow for a precise identification of each isomer, while the ratio between the two peak currents (at the same concentration) can be large enough to such a purpose. This means that it is only possible to calculate the enantiomeric excess for an enantiomeric mixture using a calibration curve at a certain concentration. A predetermination of the total amount of analyte is thus necessary. Although one more passage is thus necessary (the determination of the total amount of the racemic analyte), the accuracy with which the enantiomeric excess is determined can be high, thus avoiding the need for more specialized and expensive equipment (e.g., chiral HPLC).

As a general comment, it is clear that the research on chiral CDs is still in its infancy. While the influence of different synthetic parameters, including a variation in ligands and solvents, temperature, and reaction time, on the properties of chiral CDs have already been investigated, more research is needed to clarify the corresponding physical and chemical mechanisms of these phenomena. 

In addition, one of the most fundamental issues is the lack of systematic and scalable synthetic procedures to produce high-quality chiral CDs with desired structures such as size, shape, crystallinity, functional groups, defect types and positions. The relative exact reaction mechanisms and chiral transfer patterns are unclear for all these reasons. Nonetheless, in some cases, a large-scale synthesis is possible, paving the way to more extensive applications, not only in the sensing field [57].

Next, it is still a challenge to develop practical chiral sensing with portable sensors. Therefore, portable and stable sensors for chiral analysis should be produced for quantitative detection in situ, which is also a key factor for chiral sensing before its commercial applications start.

Finally, if intelligent chiral sensors are designed, integration of nanomaterials such as chiral CDs with appropriate chiral selectors is required, paving the way towards commercialization. In fact, the large-scale use of carbon dots (chiral or not) in whatever application field passes through their large-scale synthesis, which, in many cases, is still not possible. Nonetheless, the established syntheses lead to quite reproducible nanoparticles regarding both dimensions and physico-chemical properties. We believe the good results obtained in their applications (sensors or others) will spur efforts in their large-scale synthesis optimization.

## Figures and Tables

**Table 1:** Fluorescent/colorimetric sensors for enantiorecognition based on carbon dots *.

Entry	CD Synthesis	Chiral Agent	Optical Technique	Analyte/ Sample	LR	LOD	Ref.
1	Hydrothermal, OTD/L-Trp	L-Trp	F/C	L/D-Gln, L/D-Val/-	0.1–5 mM	1.30 μM	[33]
2	Solvothermal, GdCl_3_/PEI/DH	no	F/C	L/D-Glu/human urine, mouse serum	0–1.50 mM	3.90 μM	[34]
3	Hydrothermal, vine tea/NADES	D-Fructose	F	L-Lys/urine, human serum, energy drinks	0–3 mM	10 nM	[35]
4	Thermal, L-Glu	L-Glu	F	L/D-Leu/body building supplements, human serum	0–1.14 μM	1.7 nM	[36]
5	Hydrothermal, CA/Cys	GOX	F/C	D-Glucose/blood	0–12 mM	0.1 mM	[37]
6	Microwave, CA/urea	DAAO	F	D-Ala/human serum, artificial gastric fluid, BGC-823 cells	0–100 mM	0.55 mM	[38]
7	Hydrothermal, CA/L-Asp	L-Asp	F	L/D-Lys/-	0–1.0 mM	3.44 μM	[39]
8	Hydrothermal, CA/4-pyridinemetha- neamine	(1*S*,2*S*)-1,2- diaminocyclohexane	F	R/S-ABO R/S-APO/-	3.0–5.0 mM		[40]
9	Thermal, CA/ethylenediamine	L-Cys	F	L-Lys/-	0–5.0 mM	0.97 mM	[41]
10	Thermal, CA	(1*R*,2*R*)-1,2- diaminocyclohexane	F	L/D-PA R/S-PE/-	0–0.1 mM		[42]
11	Hydrothermal, CA/L-Cys	L-Cys	F	L/D-FA/FA supplements	0–0.33 mM	0.31 μM	[43]
12	Hydrothermal, Folic acid/PEI	Folic acid	F	L/D-Cys/-			[44]
13	Hydrothermal, BSA/metal cation	BSA	C	L/D-Tyr, His, Thr, Leu, Cys/-	0.5–20 mM	1 μM	[45]

* Table 1 legend: F: Fluorescence; F/C: Fluorescence and Colorimetric; C: Colorimetric; LOD: Limit of detection; LR: Linear range; ABO: 2-Aminobutan-1-ol; APO: 2-Aminopropan-1-ol; BSA: Bovine serum albumin; BCC-823: B gastric cancer-823CA: Citric acid; DAAO: D-@-Amino acid oxidase; DH: Dopamine hydrochloride; Gox: Glucose oxidase; NADES: Natural deep eutectic solvent; OTD: *N*-methyl-1,2-benzenediamine; PA: Phenylalaninol; PE: Phenylethanol; PEI: Polyetherimide; Ala: Alanine; Asp: Aspartic acid; Cys: Cysteine; Gln: Glutamine; Glu: Glutamic acid; His: Histidine; Leu: Leucine; Lys: Lysine; Thr: Threonine; Trp: Tryptophan; Tyr: Tyrosine; Val: Valine.

**Table 2:** Electrochemical sensors for enantiorecognition based on carbon dots *.

Entry	CD Synthesis	Chiral Agent	Electrochemical Technique	Analyte/ Sample	I_L_/I_D_	LR	LOD	Ref.
1	Thermal, CA	CS	DPV	L/D-Trp/-	2.06			[47]
2	Hydrothermal, CA/EDA	CS	DPV	L/D-Trp/-	2.28	1–6 mM		[48]
3	Hydrothermal, L/D-Cys	L/D-Cys	EIS/LSV	L/D-Tart/-	1.3			[29]
4	Thermal, melamine	CMF	PEC	L/D-Thyroxine/ Serum, commercial tablets	1.38	0.1–10 nM	67–85 pM	[49]
5	MW pyrolysis, sorbitol	Sorbitol	CV	L/D-Tyr/-	1.62	0.2–1.2 mM		[50]
6	Hydrothermal, CA/EDA	β-Cyclodextrin	DPV	L/D-Trp/-	0.90	5.0–70.0 μM		[51]
7	Acid oxidation, RGO	β-Cyclodextrin	DPV	L/D-Trp/-	2.57			[52]
8	Acid oxidation, graphene	β-Cyclodextrin	DPV	L/D-Trp/-	0.60	1.0–30.0 μM	0.12 μM	[53]
9	Acid oxidation, graphene	β-Cyclodextrin	CV	L/D-Tyr/ serum	2.35	0.0–1.5 mM 6–100 μM	6.07 nM	[54]

* Table 2 legend: CV: Cyclic voltammetry; DPV: Differential pulse voltammetry; EIS: Electrochemical impedance spectroscopy; LSV: Linear sweep voltammetry; PEC: Photo-electrochemical; I_L_/I_D_: L and D enantiomers peak current ratio; LOD: Limit of detection; LR: Linear range; MW: microwave; CA: Citric acid; CMF: chiral multifarene; CS: Chitosan; Cys: Cysteine; EDA: Ethylenediamine; GO: Graphene oxide; RGO: Reduced graphene oxide; Tart: Tartaric acid; Trp: Tryptophan; Tyr: Tyrosine.
